# An Overview: The Toxicity of *Ageratina adenophora* on Animals and Its Possible Interventions

**DOI:** 10.3390/ijms222111581

**Published:** 2021-10-27

**Authors:** Zhihua Ren, Samuel Kumi Okyere, Juan Wen, Lei Xie, Yujing Cui, Shu Wang, Jianchen Wang, Suizhong Cao, Liuhong Shen, Xiaoping Ma, Shumin Yu, Junliang Deng, Yanchun Hu

**Affiliations:** Key Laboratory of Animal Diseases and Environmental Hazards of Sichuan Province, College of Veterinary Medicine, Sichuan Agricultural University, Chengdu 611130, China; zhihua_ren@126.com (Z.R.); samuel20okyere@gmail.com (S.K.O.); juanwen881010@163.com (J.W.); wsxielei@gmail.com (L.X.); yjchoi@163.com (Y.C.); shuw0326@163.com (S.W.); wangjianchen01@163.com (J.W.); suizhongcao@126.com (S.C.); shenlh@sicau.edu.cn (L.S.); mxp886@sicau.edu.cn (X.M.); yayushumin@163.com (S.Y.); dengjl213@126.com (J.D.)

**Keywords:** *Ageratina adenophora*, toxicity, mechanisms, possible interventions, antioxidant, anti-inflammation, probiotics

## Abstract

*Ageratina adenophora* is one of the major invasive weeds that causes instability of the ecosystem. Research has reported that *A. adenophora* produces allelochemicals that inhibit the growth and development of food crops, and also contain some toxic compounds that cause toxicity to animals that consume it. Over the past decades, studies on the identification of major toxic compounds of *A. adenophora* and their toxic molecular mechanisms have been reported. In addition, weed control interventions, such as herbicides application, was employed to reduce the spread of *A. adenophora*. However, the development of therapeutic and prophylactic measures to treat the various *A. adenophora*—induced toxicities, such as hepatotoxicity, splenotoxicity and other related disorders, have not been established to date. The main toxic pathogenesis of *A. adenophora* is oxidative stress and inflammation. However, numerous studies have verified that some extracts and secondary metabolites isolated from *A. adenophora* possess anti-oxidation and anti-inflammation activities, which implies that these extracts can relieve toxicity and aid in the development of drug or feed supplements to treat poisoning-related disorders caused by *A. adenophora*. Furthermore, beneficial bacteria isolated from rumen microbes and *A. adenophora* can degrade major toxic compounds in *A. adenophora* so as to be developed into microbial feed additives to help ameliorate toxicity mediated by *A. adenophora*. This review presents an overview of the toxic mechanisms of *A. adenophora*, provides possible therapeutic strategies that are available to mitigate the toxicity of *A. adenophora* and introduces relevant information on identifying novel prophylactic and therapeutic measures against *A. adenophora*—induced toxicity.

## 1. Introduction

*Ageratina adenophora* is one of the widely known invasive weeds that negatively affects the livestock production industry [1,2]. *A. adenophora* is highly toxic to various animals and affect multiple organs; hence, it has raised serious health concerns in many countries [3,4]. For example, a study reported that the ingestion of *A. adenophora* caused respiratory disease in horses, characterized by acute edema of the lungs, which led to death [5]. Verma et al. [6] also found that *A. adenophora* reduced digestive function and photosensitive reaction in cattle. Freeze-dried leaf powder and methanol extract of *A. adenophora* caused multiple focal parenchymal necrosis and degeneration in the liver of mice [7]. Rats fed with a basal diet containing 25% (w/w) freeze-dried *A. adenophora* leaf powder showed signs of jaundice, characterized by an elevation of plasma bilirubin, ALP, ALT and AST levels [8]. Furthermore, the toxic effects of *A. adenophora* ingestion on the liver, spleen and kidney of goat and mice have also been reported, with dose-dependent apoptosis and autophagy and disorders, such as cholestasis, bile duct hyperplasia, liver necrosis, swelling and bleeding in the spleen and kidneys [9,10,11]. In addition, ≥20% dose of *A. adenophora* increased liver weight, induced severe inflammation, increased reactive oxygen species (ROS) production, and activated pyroptosis [3]. Mice fed with 175 mg/kg *A. adenophora* extract had decreased antioxidant function by reducing the activities of SOD, CAT, and GSH, while increasing the levels of lipid peroxide (LPO) in the liver [12]. *A. adenophora* caused a disorder in the arrangement and inhibited the activities of the splenocytes and immune cells in mice [13]. This shows that *A. adenophora* induces oxidative stress in the liver, thereby damaging it [14]. 

## 2. Invasive Nature of *A. adenophora*

*A. adenophora* was first introduced into the Yunan province from the China–Burma border in 1940 [15], and eventually spread to Sichuan, Guangxi, Guizhou, Hubei and Tibet provinces, the Chonqing municipality and even to Taiwan [16]. *A. adenophora* is one of the most important invasive plant species in China [17]. It tops the list of China’s first foreign invasive species released by the State Environmental Protection Administration and the Chinese Academy of Sciences, and currently affects over 30 million hectares of arable land [18]. It is predicted to spread further northward and eastward at an average speed of 20 km/year [16]. *A. adenophora* is native to Mexico and Costa Rica and has successfully invaded habitats across the world [19,20]. *A. adenophora* is normally found in roadsides, pastures, fence lines, waste areas and riparian zones as well as urban open spaces, open woodlands and forest margins in subtropical and warmer temperate regions [21]. Its rapid spread is due to its allelopathic competition with other plant species [22]. It was first reported in Australia in 1904 and has spread along the shorelines of New South Wales and southern Queensland [23]. The plant is documented as a weed in 10 states of the United States of America. In addition, *A. adenophora* was ranked as a Class 4 Noxious Weed under the NSW Noxious Weeds Act of 1993 [24]. It was tagged as an invasive weed species, due to its wide distribution in many continents, such as Asia, Africa, America and Europe [21,25].

## 3. Major Toxins in *A. adenophora* and Their Toxic Nature

The structure and function of the liver and spleen make them highly susceptible to pathogen and toxin destruction [26]. 

**Figure 1:**
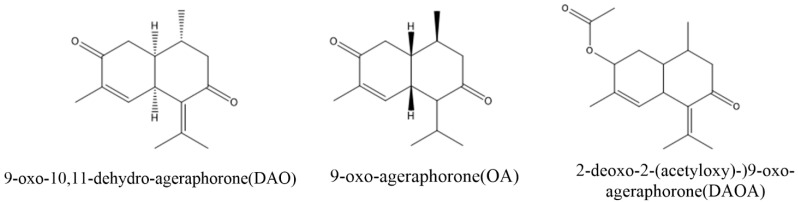
Structure of major toxins compounds in *A. adenophora*.

Numerous sesquiterpenes were identified in *A. adenophora* of which most have the same molecular skeleton as cadinene. Among these sesquiterpenes, 9-oxo-10, 11-dehydro-agerophorone (euptox A), 2-deoxo-2-(acetyloxy)-9-oxo-ageraphorone (DAOA) and 9-oxo-agerophorone (OA) are the major toxic compounds found in *A. adenophora* (Figure 1). The main differences between the molecular structure of DAOA and OA is the presence of a 2-acetoxy group in the DAOA and 2-carbonyl group in OA, whereas the distinguishing feature between euptox A and OA is the presence or absence of an unsaturated 6–11 bond in conjugation with a 7-oxo function [27]. These toxins are mainly found in the leaves with a mass percentage of 0.63–1.99 % in dry leaves [28]. 9-Oxo-10, 11-dehydro-agerophorone (euptox A) exhibited hepatotoxicity in rodents [7,29,30,31] with a median lethal dose (LD50) of 1470 mg/kg body weight of mice, whereas 2-deoxo-2-(acetyloxy)-9-oxo-ageraphorone(DAOA) and 9-oxo-agerophorone (OA) also showed hepatotoxicity in mice with respective LD50 of 926 mg/kg BW and 1470 mg/kg BW [27]. DAOA and euptox A are also immunotoxic to mice, showing characteristics such as reduction in numbers and irregular arrangement of splenocytes and thymocytes [13]. Euptox A can cause the arrest of splenocyte proliferation in the G0/G1 phage and induce autophagy in a dose-dependent manner when administered to mice via the gastric route [31]. *A. adenophora* is dangerous to animals because of its toxic nature; hence, there is the need to develop control strategies and also establish therapeutic measures to attenuate its toxicity once ingested into the body.

## 4. Molecular Mechanism of *A. adenophora* Toxicity

The exposure of animals to *A. adenophora* causes an elevation in reactive oxygen species (ROS) parameters, such as nitric oxide, superoxide and hydroxyl radicals, leading to the damage of DNA and proteins in addition to altering the cellular architecture, permeability and cell survival [32,33]. *A. adenophora* is reported to trigger a series of downstream signaling cascades and further interrupt signaling pathways associated with cell growth, proliferation and apoptosis [3]. The mechanism of ROS formation by *A. adenophora* is yet to be thoroughly decoded. However, several studies have indicated the involvement of mitochondrial complex I in *A. adenophora*–mediated oxidative stress in different types of cells. The presence of *A. adenophora* in cells results in DNA damage, due to its ROS-forming ability through interactions with oxygen [3]. *A. adenophora* also causes toxicity by reducing the mitochondria membrane potential and through the release of cytosol from the mitochondria membrane [29]. Furthermore, *A. adenophora* causes an imbalance of apoptosis-related enzymes, Bax and BCl_2_, which causes the activation of caspases, thereby resulting in DNA fragmentation and apoptosis. 

*A. adenophora* causes mitochondria dysfunction through the reduction of the cellular antioxidant systems, such as glutathione (GSH), and nicotinamide adenine dinucleotide phosphate (NADPH) levels, which may also disrupt the maintenance of the reduced states of thiol-containing proteins in the mitochondria. This causes the oxidation of the thiol-containing proteins, which in turn, changes the conformation of the mitochondrial membrane permeability transition pore, causing its opening, thereby promoting apoptosis and necrosis [34]. Therefore, at the cellular level, it can be concluded that *A. adenophora* triggers necrotic cell death, leading to multiple organ failure. 

*A*. *adenophora* can induce an extensive inflammatory response [35]. Increased levels of ROS are associated with various diseases, such as chronic inflammation [36], and this promotes the release of various pro-inflammatory factors [37]. *A. adenophora* was reported to cause pyroptosis in the spleen of mice at the dose of 10% and above [14]. Pyroptosis involves the inflammatory response of pro-inflammatory cytokines, such as caspase-1 activation and interleukin-1β (IL-1β) production [38,39,40]. Caspase-1 protease, a major constituent of the multiprotein inflammasome complexes, is involved in the activation and secretion of IL-1β, a pro-inflammatory cytokine [41]. Numerous studies have reported that pyroptosis is an immune effector mechanism that occurs in various types of cells [42,43] and is activated by diverse pathological stimuli [44,45], leading to the secretion of pro-inflammatory cytokines [46]; however, the underlying mechanism for this occurrence requires further studies.

**Figure 2:**
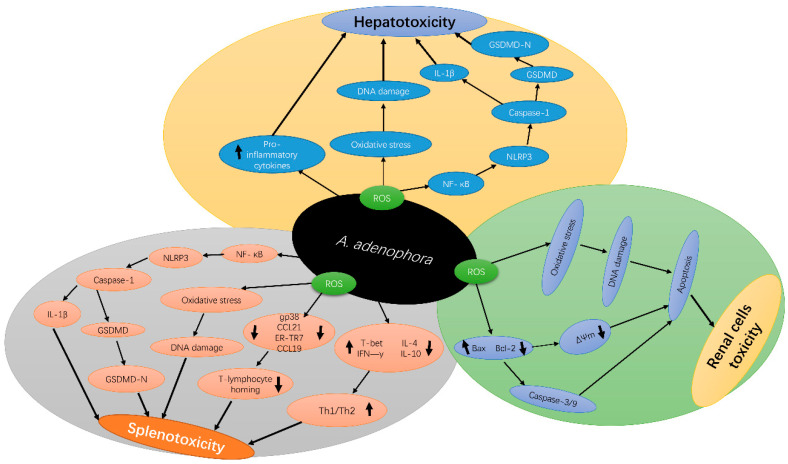
Schematic diagram of *A. adenophora*—induced toxicity in the liver, spleen and kidney and its underlying molecular mechanisms *A. adenophora* causes liver toxicity via the ROS apoptotic pathway, pro-inflammation mediated pathway, ROS-NLRP3-mediated pyroptosis pathway, and caspase-1-dependent pyroptosis pathway, *A. adenophora* causes spleen toxicity via ROS apoptotic pathway, ROS-NLRP3-mediated pyroptosis pathway, caspase-1-dependent pyroptosis pathway, destroying the Fibroblast reticulocyte (FCR) network and elevating Th1/Th2 ratio, Finally *A. adenophora* causes toxicity of the kidney via ROS apoptosis pathway, caspase 3/9 mediated pathway and mitochondria dysfunction pathway. IL-1β—Interleukin 1-beta, ROS—reactive oxygen species, GSDMD—gasdermin D, NLRP3—NOD-, LRR- and pyrin domain-containing protein 3, NF-κB—Nuclear factor-κB, Δ*Ψ*m—Mitochondria potential membrane, gp38—glycoprotein 38, Th1/2—T-helper cells 1 and 2, CCL21—C-C Motif Chemokine Ligand 21, CCL19—C-C Motif Chemokine Ligand 19, T-bet—T-box transcription factor 21, IFN-γ—Interferon-gamma, IL-4—Interleukin 4, IL-10—Interleukin 10, Bax—BCl_2_ Associated X, BCl-_2_—B-cell lymphoma-2.

Another current research reported that *A. adenophora* causes toxicity in the spleen by destroying the fibroblastic reticular cell (FRC) network and causing an imbalance in the Th1–Th2 cell ratio [33]. The study speculated that *A. adenophora* ingestion induces a persistent inflammatory response in the spleen, which in turn could lead to the activation and promotion of T cell immunity, resulting in splenic dysfunction. However, the mechanism behind these observations is not yet clear. Therefore, it requires further studies. Another study by Cui et al. [47] also reported that *A. adenophora* causes destruction of the intestinal structure and immune barrier integrity. In summary, *A. adenophora* induces inflammation in cells, which leads to cell death mediated by pyroptosis. Figure 2 depicts the molecular mechanisms of *A. adenophora*—induced toxicity in various organs.

## 5. Pharmacological Applications of *A. adenophora* and Potential Therapeutic Interventions against Its Toxicity

*A. adenophora* is used in the traditional system of medicine across the world. In India, leaves of the plant are pharmacologically regarded as astringent, thermogenic, stimulants and are used as medicine because of the antimicrobial, antiseptic, blood coagulating, analgesic, and antipyretic properties [48]. Furthermore, a decoction of the plant is recommended for treating jaundice and ulcers [49]. In Nigerian traditional medicine, it is used to treat fever, diabetes, and inflammation [50]. These pharmacological properties may be the result of bioactive secondary metabolites present in the plant. A study by Fu et al. [51] recently reported that two metabolites, phomoxanthone A and penialidin A produced by a fungal endophyte *Coniochaeta* sp. F-8, isolated from *A. adenophora*, showed antioxidant activities, and hence, had great importance in biotechnology as a source of novel bioactive compounds for antioxidant drug development. Moreover, another study reported antiviral activity of euptox A in NDV-infected chicken embryo fibroblasts (CEFs), using the MTT method [52]. The results showed that euptox A at 10 μg/mL could directly suppress, neutralize, and block NDV in vitro as well as prevent the binding of NDA to its receptor. Nong et al. [53] also reported the acaricidal activity of ethanol extract from leaves of *A. adenophora*. A study by Rajeswary et al. [54] reported that crude extracts derived from *A. adenophora* had ovicidal effects against mosquito eggs at concentrations of 300 mg/L; hence, the plant could be used for controlling mosquitos. Numerous studies have showed antimicrobial activity of *A. adenophora* and its extracts [48]. A study reported that *A. adenophora* inhibited *Phytophthora capsici* at 50–250 mg/mL concentrations [55]. Another study also reported that oils extracted from *A. adenophora* showed significant toxicity against *Erwinia herbicola* and *Pseudomonas putida*, two phyto-pathogenic bacteria at concentrations of 0.25–5 µL mL^−1^ [56]. Both organic and aqueous crude extracts from leaves of *A. adenophora* showed inhibitory effects on the growth of *Bacillus subtilis*, *Bacillus cereus* and *Pseudomonas aeruginosa* [57]. Furthermore, methanolic leaf extract from *A. adenophora* showed an obvious inhibitory effect on *Pseudomonas aeruginosa* [58]. Euptox A also showed potent effects against the widespread plant pathogen *Ralstonia solanacearum* (R1-4), with the minimum inhibitory dose ranging from 0.25 to 1 mg/mL [59]. The thymol derivatives of *A. adenophora* have shown inhibitory effects against both Gram-negative and Gram-positive bacteria [60]. 

A number of secondary metabolites isolated from the inflorescence and roots of *A. adenophora*, mainly sesquiterpenes, showed potent antifungal activity [48]. Several studies have reported the inhibitory effects of the crude extracts of *A. adenophora* against pathogenic fungi [61,62]. For example, 100.00 mg/mL *A. adenophora* ethanol, acetone, and ether extract showed 100% inhibitory rate against *Fusarium gramincarum* and *Colletotrichum glycines* Hori [63]. In addition, Liu et al. [64] reported that the leaf extracts of *A. adenophora* (mainly 10Hβ-9-oxo-agerophorone, 10Hα-9-oxo-agerophorone and euptox A) inhibited the formation of *Pythium myriotylum* mycelial biomass at the minimum inhibitory concentration of 100 μg/mL. Euptox A also inhibited germination of *Fusarium oxysporum*, *Bipolaris sorokiniana*, *Fusarium proliferatum* and *Alternaria tenuissima* as well as spore production in *Fusarium oxysporum* and *Bipolaris sorokiniana* [65]. In the latest study of Hu et al. [66], it was found that both euptox A and cadinan-3-ene-2,7-dione (CED) isolated from the methanol extract of *A. adenophora* showed antifungal activities characterized by the destruction of the integrity of cell membranes and inhibition of ergosterol synthesis, which eventually led to fungal cell death. The oil extract from *A. adenophora* inhibited the mycelial growth of *Phytophthora capsici* at the concentration of 500 μg/mL after 7 days of incubation [67]. Furthermore, in recent years, the use of *A. adenophora* as an anti-nematode agent and an insecticide was recognized. A recent study by Lin et al. [68] reported a stronger resistance of *A. adenophora* to *Aphis gossypii* feeding. The methanol extract of *A. adenophora* showed good toxicity to radish aphids, and also had a certain inhibitory effect on the growth and development of *Mythimna separata*. The acetone extract of *A. adenophora* had a toxic effect against cabbage aphids and *Brevicoryne brassicae* [69]. Similar results were observed by Wang [70] on *Aphis gossypii*. 

Furthermore, anti-cancer/tumor properties of *A. adenophora* were reported in recent studies. For example, a study by André et al. [71] reported that euptox A isolated from *A. adenophora* showed a strong potential against cancer by acting on cancer targeted cellular characteristics. Similarly, Liao et al. [72] also studied the antitumor activity of euptox A isolated from *A. adenophora* in vitro against three cell lines, using the 4,5-dimethylthiazol-2-yl)-2,5-diphenyltetrazolium bromide (MTT) assay. The results showed that euptox A had significant antitumor activity against the three tumor cell lines in vitro in a dose-dependent manner. Euptox A percentage inhibition on the human lung cancer A549 cells, Hela cells, and Hep-2 cells were 76.42%, 68.30% and 79.05%, respectively, at a concentration 500 μg/mL, whereas the 50% inhibitory concentration (IC50) of euptox A for the three tumor cell lines were 369 μg/mL, 401 μg/mL and 427 μg/mL (A549, Hela and Hep-2 cells, respectively). Another study by Chen et al. [73] reported that essential oil from *A. adenophora* promoted HCC (hepatocellular carcinoma) apoptosis by activating the mitochondria and endoplasmic reticulum apoptotic signaling pathways as well as inhibiting the action of STAT3 (signal transducer and activator of transcription 3) and AKT (protein kinase B). 

Other important pharmacological activities of *A. adenophora*, such as its anti-pyretic, analgesic and wound healing abilities, were reported recently. Ringmichon and Gopalkrishnan [74] reported that the aqueous extracts at doses of 300 and 400 mg/kg body weight showed a significant decrease in pyretic temperature a few hours after treatment, which was similar to the standard drug (paracetamol; 150 mg/kg body weight). The methanolic extract of *A. adenophora* leaves showed significant analgesic activity as compared to standard drugs, diclofenac sodium and pentazocine, in an acetic acid–induced writhing test, tail immersion test, and tail-flick test models [75]. Finally, Kumar et al. [76] investigated the wound healing properties of ethanolic extract of *A. adenophora* formulated as a gel, using the excision and incision wound models. The results showed that the gel could strongly heal the wound in excision with 90.98% wound contraction and 36.16% reduction in epithelialization time, whereas in the incision model, the gel significantly increase (37.86%) the tensile strength on the 13th day of treatment when compared to pure gel control. In a nutshell, *A. adenophora* produces bioactive compounds that exhibit pharmacological activities and therefore, could be adopted to develop potential drugs or feed supplements to prevent or treat health complications caused by *A. adenophora* toxicity. Therefore, some potential therapeutic drug candidates (plant extracts, secondary metabolites, and bacteria) from *A. adenophora* and other sources that could be used to treat or prevent the two major pathogeneses (oxidative stress and inflammation) of *A. adenophora* toxicity includes the following. 

### 5.1. Anti-Oxidant Therapeutic Candidates for A. adenophora Toxicity

Although many studies have reported on the toxic effects of *A. adenophora*, other studies have also reported on the plant’s beneficial biological activities, such as its antioxidant, anti-inflammation, anti-microbial, anti-obesity, anticancer and anti-tumor qualities [71,75,77]. These beneficial activities induced by *A. adenophora* could be attributed to the presence of bioactive compounds in this plant [22]. Oxidative stress is one of the major pathogeneses for *A. adenophora* toxicity; therefore, the use of antioxidants, especially from natural products, could help eliminate the toxic effects induced by this plant. Numerous bioactive extracts and secondary metabolites in *A. adenophora* were reported to possess antioxidant properties. For example, ethanolic extract from the leaves of *A. adenophora* was reported to reduce the generation of hydroxyl radicals [22]. Furthermore, the quinic acid derivative, including 5-O-trans-o-coumaroylquinic acid methyl ester, chlorogenic acid methyl ester, macranthoin F and macranthoin G isolated from the leaves of the plant, showed antioxidant activity against DPPH (1, 1-diphenyl-2-picrylhydrazyl) radical [78]. Another study that used 2, 2-diphenyl-1-picrylhydrazyl (DPPH) radical scavenging protocol and the ferric reducing ability assay (FRAP) reported that essential oils and cadences extracted from the leaves of *A. adenophora* showed antioxidant activity similar to the test standards [12]. In addition, oil extracts from *A. adenophora* showed antioxidant activity, with IC50 values of 8.3 and 4.2 µL, after being tested using the DPPH and β-carotene bleaching methods, respectively [22]. Lastly, methanolic extracts from *A. adenophora* showed significant DPPH activity as compared to the standard butylated hydroxyl toluene (IC50 for *A. adenophora* was 92.791, whereas that for butylated hydroxyl toluene was 68.043) [79]. Therefore, harnessing these extracts and secondary metabolites into antioxidant drugs or feed supplements to reduce the ROS damages induced by *A. adenophora* and other toxins could play an important role in reducing the toxicity of this plant as well utilizing the plant’s resources for the benefit of mankind. However, even though various studies have reported the antioxidant properties of some extracts and secondary metabolites from *A. adenophora*, there is still the need for effective clinical studies and monitoring to ascertain the safest dose concentration and periods for administration before drug development. Other potential antioxidant agents that could be adopted to reduce the oxidative stress mediated toxicity induced by *A. adenophora* are shown in Table 1.

### 5.2. Anti-Inflammatory Therapeutic Candidates for A. adenophora Toxicity

As already established, inflammation remains one of the main pathogeneses of *A. adenophora* toxicity in cells. Therefore, the adoption and use of anti-inflammatory products to treat the toxicity of *A. adenophora* and its derivatives is a promising strategy for reducing the health complications or death in animals who have ingested this plant. The anti-inflammatory activity of extracts and secondary metabolites of *A. adenophora* were reported in various studies. For example, the ethanolic leaf extract of *A. adenophora* showed anti-inflammatory activity via the inhibition of IL1β and cyclooxygenase 2 (COX-2) genes [22]. In addition, a study reported that the intravenous administration of the leaf extract of *A. adenophora* increased the number of CD4^+^ T cells in the spleen, induced TGFβ encoding (a cytokine involved in tissue repair mechanism), and inhibited the expression of IL1β and COX-2 genes responsible for the metabolism of inflammatory mediators [91,92]. Furthermore, ethanolic extracts from the leaves of *A. adenophora* showed an anti-inflammatory role via the inhibition of hydroxyl radical generation [22,91]. In addition, the ethanolic leaf extract of *A. adenophora* was reported to suppress efficiently the inflammatory reaction set in foot paw induced by injecting dinitrofluorobenzene (DNFB) [92]. Therefore, the effective development of anti-inflammation drugs from these extracts and secondary metabolites could be a novel clinical strategy to mitigate the toxic effects of *A. adenophora* exposure. However, safe doses and administration periods require thorough research. In addition to extracts and secondary metabolites extracted from *A. adenophora* that have anti-inflammatory properties, other natural products that have demonstrated anti-inflammatory activities are shown in Table 1. 

### 5.3. Degrading Microbes and Probiotics Therapeutic Candidates for A. adenophora Toxicity

Micro-organisms are indispensable to the nutrition and wellbeing of the host, including humans and animals [93]. Some of these microorganisms have been reported to degrade various toxic compounds in food, soil and the environment. For example, some strains of *Pseudomonas*, *Acinetobacter*, *Mycobacterium*, *Haemophilus*, *Rhodococcus*, *Paenibacillus*, and *Ralstonia* were reported to encompass the metabolic pathways required for the degradation of many hydrocarbons and mycotoxins [94,95,96]. Similarly, some bacteria were also identified to be able to degrade some major toxins of *A. adenophora. Stenotrophmonas* spp isolated from *A. adenophora* could degrade euptox A, thus making *A. adenophora* safe to feed livestock [72]. Furthermore, a more recent study reported that tannase-producing rumen bacteria, *Klebsiella variicola* strain PLP G-17 LC, *Klebsiella variicola* strain PLP S-18 and *Klebsiella* pneumonia strain PLP G-17 SC could also degrade euptox A. These findings suggest that using the above microbial strains as microbial feed supplements could enhance the utilization of *A. adenophora* to alleviate the toxicity caused by euptox A (*A. adenophora*). Therefore, there is a need for further studies to isolate more such beneficial bacteria to help degrade the other main toxins (such as 2-deoxo-2-(acetyloxy)-9-oxo-ageraphorone and 9-oxo-agerophorone) in the plant. These bacteria and fungi could be developed into probiotics or other feed supplements that would be administered to animals that are highly exposed to *A. adenophora* and other noxious plants to prevent or reduce their toxicity. However, to achieve this, there is the need to investigate the efficacy and safety of these microbial strains through standardized experimental animal feeding trials. 

Another promising therapeutic intervention for the treatment of toxicity caused by *A. adenophora* that has not been tested yet is the administration of probiotics. Probiotics have been reported to improve the antioxidant status and reduce inflammation in most animal species [97,98]. Additionally, numerous studies have reported the protective effect of probiotics in oxidative stress and inflammation induced by various toxins. For example, *Lactobacillus* spp was reported to reduce oxidative stress induced by deoxynivalenol (DON) via reducing the production of ROS in broiler chicken [99]. In addition, *Lactobacillus salivarius* BP121 was reported to decrease the inflammation and oxidative stress in cisplatin-induced acute kidney injury in rats [100]. Therefore, there is the need for advanced studies on the effects of various types of probiotic strains on the toxicity induced by *A. adenophora* to effectively understand the molecular bases for the treatment of *A. adenophora*–induced toxicity by probiotics.

## 6. Discussion and Future Prospects

Over the past years, conscious efforts have been put in place to reduce the growth and spread of *A. adenophora*. Various weed control strategies have been established to control the spread of this plant to reduce its toxic effects on the environment; however, due to the plant’s growth patterns and invasive nature, all these efforts have not yielded good results so far [4]. In addition, lacking in strategies for the plants’ elimination, scientists have only focused on the development of strategies to reduce the spread and distribution of this plant and the plants toxicity, without giving much attention to the therapeutics after ingestion of this plant. 

Plants and plant products are used to treat numerous diseases, as they continue to serve as a potent source of new medicinal candidates, and for the treatment of emerging diseases. For example, a study by Fernández et al. [101] reported that flavonoids could provide a dual effect for the combination treatment, potentiating the antitumor effect of 5-FU, and concurrently, avert important side effects of 5-FU chemotherapy. In addition, another study reported that administration of phytocannabinoids isolated from *Cannabis sativa* improves the health and function of the gastrointestinal tract [102]. Freitas et al. [103] also reported that *Spirulina platensis* is a safe natural analgesic that displays great therapeutic activity in inflammatory pain disorders. Therefore, this review revealed extensive research on some extracts and secondary metabolites extracted from *A. adenophora* and other sources that could be used for the treatment of toxicity induced by *A. adenophora* through intensive investigations and clinical trials. Furthermore, a major section of this review highlighted the antioxidant and anti-inflammatory properties of the extracts, secondary metabolites and other agents that could counter the oxidative stress and inflammation-mediated toxic effects induced by *A. adenophora* exposure. In addition, this paper revealed some beneficial bacteria that were reported to degrade some major toxic molecules released by *A. adenophora*, and suggested the adoption of probiotics in treating *A. adenophora* toxicity as a promising therapeutic strategy since numerous probiotic strains have been reported to have antioxidant and anti-inflammatory properties [97]. In summary, this review seeks to bring awareness to the scientific community on the potential utilization of *A. adenophora* plant resources (extracts, secondary metabolites and endophytes) and other promising agents as useful products (such as dietary supplements or drug candidates) to prevent or treat the toxic effects associated with *A. adenophora* and other toxic plant intoxication. This field remains wide open for exploration of natural product formulations and genetic manipulations that can not only offer protection against *A. adenophora*–mediated toxicity, but also can serve as a therapeutic measure to reverse the toxic effects induced by *A. adenophora*.

## Figures and Tables

**Table 1:** Potential antioxidant and anti-inflammatory agents for treatment of *A. adenophora*–induced toxicity.

	Antioxidant Agents	Animal Model	Dosage	Activities	Reference
1	Quercetin and vitamin E combination	Chicken	0.4 g/kg and 0.2 g/kg respectively for 10 weeks	Reduce ROS Increase total antioxidant capacity (T-AOC) Reduce pro-inflammation cytokines	[80]
2	Resveratrol	Mice	40 mg/kg for 6 months	Reduce ROS Reduce pro-inflammation cytokines	[81]
3	Lycopene	Rat	10 and 20 mg/kg for 30 days	Reduce ROS Reduce pro-inflammation cytokines (IL-6, IL-1β, TNF-α)	[82]
4	Glycine Nano-selenium	Rats	0.05 and 0.1 mg/kg for 30 days	Decrease the MDA levels	[83]
5	Alfalfa saponins	IEC-6 cells	75, 100, 150, 200 and 300 μmol/L for 24 h	Elevate the amount of T-AOC in cells	[84]
6	Malus doumeri leaf flavonoids	human embryonic kidney 293 T cells	160 μg/mL for 48 h	Increase the levels of catalase (CAT), superoxide dismutase (SOD), glutathione (GSH), and glutathione peroxidase (GSH-Px) and reduce the level of malondialdehyde (MDA)	[85]
7	Oregano essential oil	RAW264.7 Cells	2.5–10 μg/mL for 24 h	Inhibited the mRNA expression of IL-1β, IL-6, and TNF-α in the RAW264.7 cells	[86]
8	Ergosterol	16 HBE cells and Balb/c mice	5, 10 and 20 μM for 24 h and 40 mg/kg for 21 days	Decrease the expression of interleukin-6 (IL-6), tumor necrosis factor α (TNF-α),	[87]
9	Ginger	Pulmonary TB patients (human)	3 g of ginger extract daily for 1 month	Reduced the levels of tumor necrosis factor (TNF) alpha	[88]
10	Selenium	Chicken	1 mg/kg for 12 weeks	Reduced the levels of inflammation-related factors (Nuclear factor-kappa B, tumor necrosis factor-α, cyclooxygenase-2, NLRP3, apoptosis-associated speck-like protein containing a caspase recruitment domain, caspase-1, interleukin (IL)-1β, IL-6, IL-18 and interferon-γ)	[89]
11	Probiotics (*Lactobacillus acidophilus*, *Lactobacillus casei*, *Lactococcus lactis*, *Lactobacillus reuteri*, and *Saccharomyces boulardii*)	Human colon epithelial HT-29 cells	10^8^ CFU/mL for 18 h	Reduce IL-1β, IL-6, TNF-α, and increase IL-10 production Increased % of DPPH scavenging activity	[90]

## Data Availability

Not applicable.
